# A novel *CUL4B* gene variant activating Wnt4/β-catenin signal pathway to karyotype 46, XY female with disorders of sex development

**DOI:** 10.1186/s40659-024-00583-1

**Published:** 2025-01-07

**Authors:** Chunlin Wang, Hong Chen, Qingqing Chen, Yangbin Qu, Ke Yuan, Li Liang, Qingfeng Yan

**Affiliations:** 1 Department of Pediatrics, The First Affiliated Hospital of Zhejiang University School of Medicine, Hangzhou, China; 2 Department of Endocrinology, Fuzhou Children’s Hospital of Fujian Medical University, Fuzhou, Fujian China; 3 College of Life Sciences, Zhejiang University, Hangzhou, Zhejiang China; 4 Key Laboratory for Cell and Gene Engineering of Zhejiang Province, Hangzhou, Zhejiang China

**Keywords:** *CUL4B*, Disorders of sex development, Complete gonadal dysplasia, Gene, Wnt4/β-catenin

## Abstract

**Background:**

Karyotype 46, XY female disorders of sex development (46, XY female DSD) are congenital conditions due to irregular gonadal development or androgen synthesis or function issues. Genes significantly influence DSD; however, the underlying mechanisms remain unclear. This study identified a Chinese family with 46, XY female DSD due to the *CUL4B* gene.

**Methods:**

The proband medical history and pedigree were investigated. Whole-exome sequencing was performed to analyze different variations. Transiently transfected testicular teratoma (NT2/D1), KGN ovarian cells with either mutant or wild-type *CUL4B* gene, and knock-in *Cul4b* mouse models were confirmed. The expression levels of sex-related genes were analyzed.

**Results:**

A 9.5-year-old girl was diagnosed with 46, XY DSD. A hemizygous variant c.838 T > A of the *CUL4B* gene was detected. The mRNA and protein levels of *WNT4* and *FOXL2* genes were higher than those in the wild-type group; however, *CTNNB1*, *SOX9,* and DMRT1 were lower in the wild-type group in NT2/D1 cells. In KGN ovarian cells of the mutant group, the mRNA and protein levels for *WNT4* and *CTNNB1* were elevated. Damaged testicular vasculature and underdeveloped seminal vesicles were observed in *Cul4b*^L337M^ mice.

**Conclusions:**

A missense *CUL4B* variant c.838 T > A associated with 46, XY female DSD was identified, and may activate the Wnt4/β-catenin pathway. Our findings provide novel insights into the molecular mechanisms of 46, XY female DSD.

**Supplementary Information:**

The online version contains supplementary material available at 10.1186/s40659-024-00583-1.

## Introduction

Karyotype 46, XY female disorders of sex development (46, XY female DSD) are congenital conditions resulting from atypical gonadal development. This can be due to conditions, including Swyer syndrome (also known as complete gonadal dysgenesis) or issues with the production or function of androgens. Variation or abnormal expression of testicular or ovarian development genes is one of the primary causes of 46, XY female DSD. Whole-exome sequencing (WES) is the standard clinical examination for investigating known or novel genes associated with DSD. However, only 13% to 43% of DSD patients with specific disease-causing genes (including *SRY, AR, SRD5A2,* and *SF1*) have been identified [1–3]. Although an increasing number of novel causative genes are being discovered, their specific roles in the network of gonadal development remain unclear.

The development of the gonads is a dynamic and sophisticated process orchestrated by complex regulatory networks with multiple genes involved. SRY is expressed in the somatic gonad of XY individuals at the onset of the sex-determination period, upregulates SOX9, and results in the formation of testes. SOX9 promotes normal testicular differentiation and inhibits ovarian pathways through fibroblast growth factor and its receptors (FGF9/FGFR2) and testicular regulators, including double sex and mab-3-related transcription factor 1 (DMRT1) [4–7]. If the SRY/SOX9 pathway is not activated, factors including WNT family member 4 (Wnt4), β-catenin, and forkhead box protein L2 (FOXL2) promote the development of the primordial gonad to the ovary and inhibit the development of testis [8].

The Cullin 4B protein (CUL4B, encoded by the *CUL4B* gene, OMIM*300304) belongs to the cullin family. It is a scaffold protein forming the RING ubiquitin E3 ligase complex. Mutations of the *CUL4B* ubiquitin ligase gene are causally associated with syndromic X-linked mental retardation. Recently, it was reported that variants of *CUL4B* in the human germline could cause intellectual disability, short stature, central obesity, and other abnormalities in humans. Affected 46, XY individuals are frequently associated with reproductive tract abnormalities, including undescended and/or small testes, hypospadias, and small penis. *CUL4B* is reportedly involved in spermatogenesis through ubiquitination modification [9, 10]. However, information regarding the 46, XY DSD caused by the *CUL4B* genetic variants remains limited. Existing studies cannot explain the DSD phenotype in male patients with *CUL4B* gene variants.

This study analyzed clinical features, laboratory results, ultrasonographic data, and surgical and pathological reports for a girl with 46, XY female DSD. Sequencing data on the *CUL4B* gene revealed a missense variant, c.838 T > A. (p.L280M). Functional assays were performed to elucidate pathogenic mechanisms associated with the *CUL4B* variant.

## Materials and methods

### Clinical evaluations

The patient’s past medical history and symptoms were evaluated. Additionally, a physical examination, particularly of the external genitalia, was conducted. Following the laparoscopy, laboratory tests were performed, including hormonal analysis, karyotypic analysis, imagological examinations, and gonadal biopsy with histopathology.

### Cytogenetic and molecular studies

Standard techniques were used for karyotype analysis. Using standard methods, genomic DNA was extracted from peripheral blood leukocytes of the proband and her parents. Subsequently, *trio*-WES was performed following the guidelines outlined in the NCBI record NG_009388.1 (NM_001079872.2) [11, 12]. The Illumina HiSeq platform was used for WES following the manufacturer’s guidelines. Human Genome Variation Society (HGVS) nomenclature was used to name the variants [13].

### Analysis of the variant’s conservation and pathogenicity

Multiple CUL4B protein sequences were downloaded from the National Center for Biotechnology Information (NCBI). ClustalX was used for sequence alignment, and results were visualized online using ConSurf (http://consurf.tau.ac.il/) [14].

### Molecular modeling

To investigate the effect of the identified mutations on protein conformation, the 3D arrangement of human *CUL4B* (PDB code 4A0C) was used [15]. For structural representation, PyMOL 2.5 (http://www.schrodinger.com/pymol/) was used as the molecular visualization system. The interactions between amino acid residues were analyzed using DynaMut (http://biosig.unimelb.edu.au/dynamut/) [16] and Project HOPE (https://www3.cmbi.umcn.nl/hope/) [17].

### Plasmid construction

Human *CUL4B* complementary DNA (cDNA) (NM_003588) was cloned into a pCDH-CMV-MCS-EF1-Puro vector. The c.838 T > A variation was introduced into the *CUL4B* sequence through seamless cloning (Tsingke, China).

### Cell culture of HEK293T, NT2/D1, and KGN cells

HEK293T cells (ATCC^®^ CRL-11268) and NT2/D1 cells (ATCC^®^ CRL-1973TM) were cultured in HG-DMEM (Hyclone^®^) containing 10% fetal bovine serum. A granulosa cell tumor line (KGN, RIKEN Cell Bank^®^ RCB1154) of ovarian origin was cultured in DMEM/F12 medium containing 10% FBS, 1% penicillin–streptomycin, and 5% CO_2_ at 37 °C. NT2/D1 and KGN cells stably overexpressing the *CUL4B* gene were constructed by lentivirus infection. Cells were infected with lentivirus for 72 h and then used in subsequent experiments, wherein lentiviral transfection efficiency exceeded 95%.

### Quantitative real-time PCR

Cells were harvested, and total RNA was extracted using Trizol reagent and reverse transcribed into cDNA using HRbio^™^ III 1st Strand cDNA Synthesis Kit (Heruibio, China, Cat# HRF0191). The primers are listed in Table S1.

### Western blot analysis

Western blots were performed as previously described [11]. Antibodies against Phospho-β-catenin (p-β-catenin) (Cat#5961), β-catenin (Cat#8480), SOX9 (Cat#82630 T), and FLAG-tag (Cat#14,793) were procured from Cell Signaling Technology (Beverley, MA, USA). Abcam (Shanghai, China) provided Wnt4 (Cat#ab91226), FOXL2 (Cat#ab188584), DMRT1 (Cat#ab126741), and α-Tublin (Cat#ab52866).

### Development of ***Cul4b***^L337M^ mouse model

The *CUL4B* gene is highly conserved over evolutionary time. Mouse *Cul4b*^L337M^ is a homozygous variant of human *CUL4B*^L280M^. To investigate the role of the L280M mutant in vivo, we established a mouse model for precise editing of the *CUL4B* gene using the CRISPR-Cas9 system combined with fertilized egg microinjection. Briefly, the gRNA of the *CUL4B* gene (TCAAAAAGATCGATAGATGC) was designed to construct a homologous recombinant vector. The fertilized eggs of C57BL/6 J mice were injected with Cas9, gRNA, and a homologous recombination vector. The fertilized eggs that survived the injection were transplanted into pseudo-pregnant female mice, which were allowed to conceive and bear offspring. Genome DNA was extracted from the F0 generation mice born from the recipient mice for genotype identification, PCR, and sequencing. Zhejiang University Animal Ethics Committee granted animal ethics approval.

### Histology

To investigate the role of the L280M mutant in vivo, mouse testis was harvested after male mice were sacrificed. The entire testis was immersed in modified Bouin’s fixative solution for 16 h at 4 °C. After washing with PBS, the samples were then dehydrated in increasing concentrations of ethanol before being encased in paraffin wax. The sections were stained using hematoxylin and eosin (HE) and placed on glass slides.

### Statistical analyses

The western blot images were analyzed using the Fiji/Image J program. GraphPad Prism software (version 8) was used for statistical analysis. Statistical significance was determined through various t-tests, including paired, independent, and Welch’s t-tests. All findings were confirmed by a minimum of three separate experiments. Data are expressed as the mean ± standard deviation (SD) with a sample size of at least three.

## Results

### Clinical evaluation

A 9.5-year-old girl was hospitalized due to growth retardation for five years. Her height and body weight were 126.7 cm (– 1.88 SD) and 39 kg (+ 3 SD), and her BMI was 24.3 kg/m^2^. She was observed to have mild obesity and no distinguishing facial features. Her chest and pubic hair were evaluated as Tanner stage I. She had no pubic hairs or axillary hairs. External genitalia appeared typical female with no clitoromegaly. The size of the right ovary was about 0.75 × 0.63 cm, and the size of the left ovary was 0.73 × 0.38 cm. Pelvic ultrasound revealed that the uterus was about 1.45 × 0.76 × 0.97 cm, the endometrial line was unclear, the endometrial thickness was about 0.11 cm, and the muscle layer was echogenic. There were no abnormal findings in the serum adrenal hormone levels. The blood testosterone and estradiol levels were low, and blood gonadotropin levels were elevated. Table 1 summarizes the hormonal traits of the proband.

**Table 1:** Blood biochemical and hormonal characteristics of the proband

Items	Patient	Normal value
ATCH	15.9	0.00–46.00 pg/mL
LH	29.1↑	0.33–6.10 mIU/mL
FSH	2.96	1.37–6.97 mIU/mL
Progesterone	＜0.21↓	0.4–1.2 ng/ml
Testosterone	14.4	14.0–76.0 ng/dl
Estradiol	＜11.8↓	37.25–85.8 pg/ml
Cortisol_8:00am_	10.20	5.00–25.00 ug/dL

*ACTH* adrenocorticotropic hormone, *LH* luteinizing hormone, *FSH* follicularstimulating hormone

**Fig. 1:**
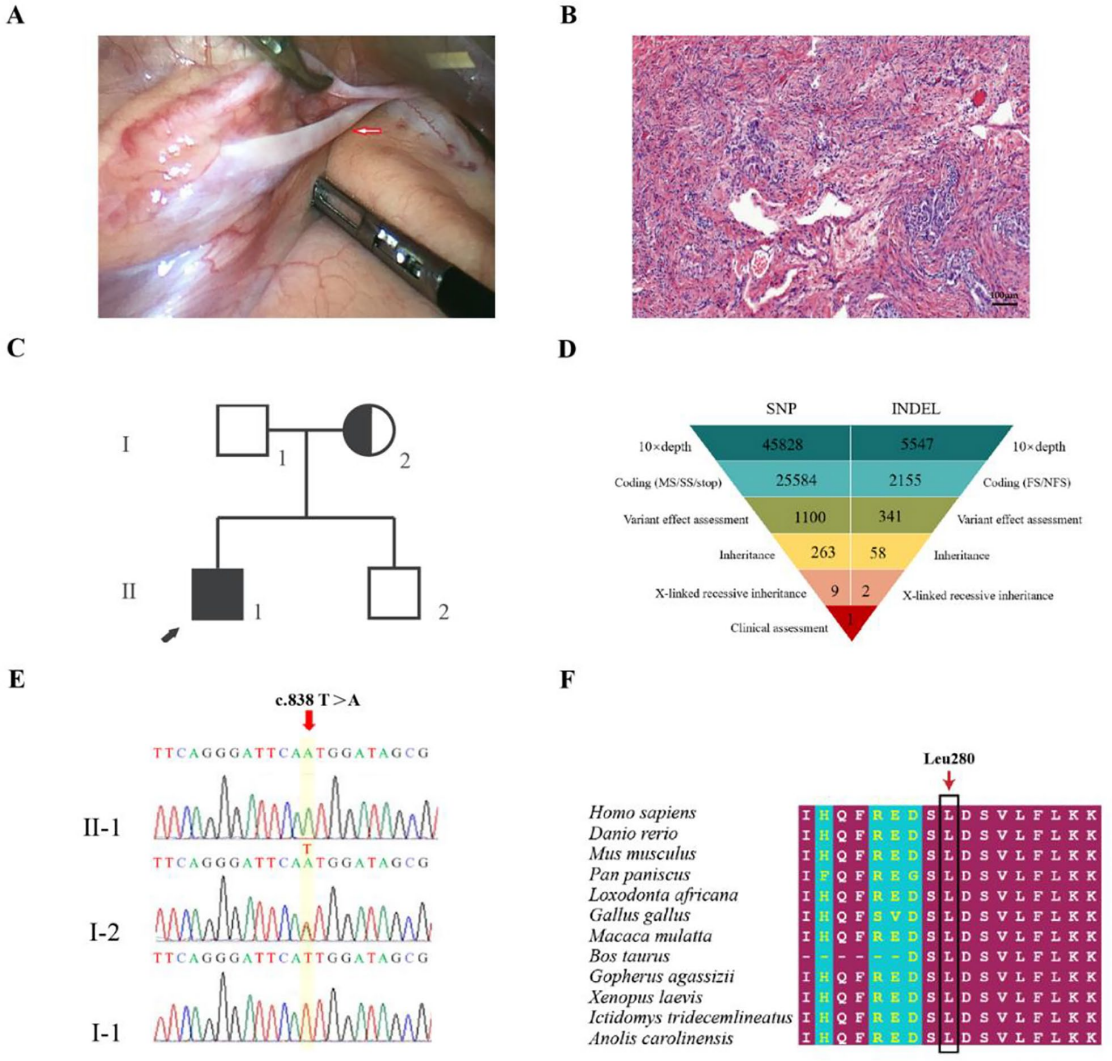
Pathologic and genetic testing of the proband. **A** Streak gonads are below the bilateral fallopian tubes (1.0 × 0.5 × 0.2 cm). **B** Pathological section analysis of proband (HE, 40 ×). Pathological evaluation revealed that the bilateral streak gonads were ovarian interstitial-like cells and fibrous-like tissue. **C** The pedigree with 46, XY female DSD. The arrowhead denotes the proband. Squares represent XY individuals, and circles represent XX individuals. Affected individuals are indicated as filled black symbols. Dot-filled symbols represent the unaffected heterozygous carriers. Unfilled symbols indicate clinically unaffected subjects harboring the wild-type sequence. **D** Schematic representation of the exome-data-filtering approach assumes dominant inheritance in the family. The following abbreviations are used: MS, missense variant; SS, splice-site variant; stop, stop-codon variant; FS, frameshift indel; and NFS, non-frameshift indel. **E** Sanger sequencing chromatograms exhibit that the proband (II-1) carried the c.838 T > A (p.L280M) hemizygous variant, and her mother (I-2) harbored a heterozygous variant of the *CUL4B* gene. **F** Interspecific conservation analysis of *CUL4B*. Color code bars are used to indicate the conservation of amino acids

Further diagnostic tests revealed a 46, XY karyotype. A laparoscopic bilateral gonadectomy was performed following genetic testing. Intraoperatively, a 1.5 × 0.7 × 0.5 cm streak of uterus-like tissue was observed. The bilateral fallopian tubes were thin, and no significant abnormalities were present at the umbilical ends of the fallopian tubes. Streak gonads were found below the bilateral fallopian tubes, and the size was about 1.0 × 0.5 × 0.2 cm. Based on pathological analysis, the bilateral streak gonads comprised ovarian interstitial cells and fibrous tissue without malignant transformation (Fig. 1A, B).

### Genetic diagnoses

**Fig. 2:**
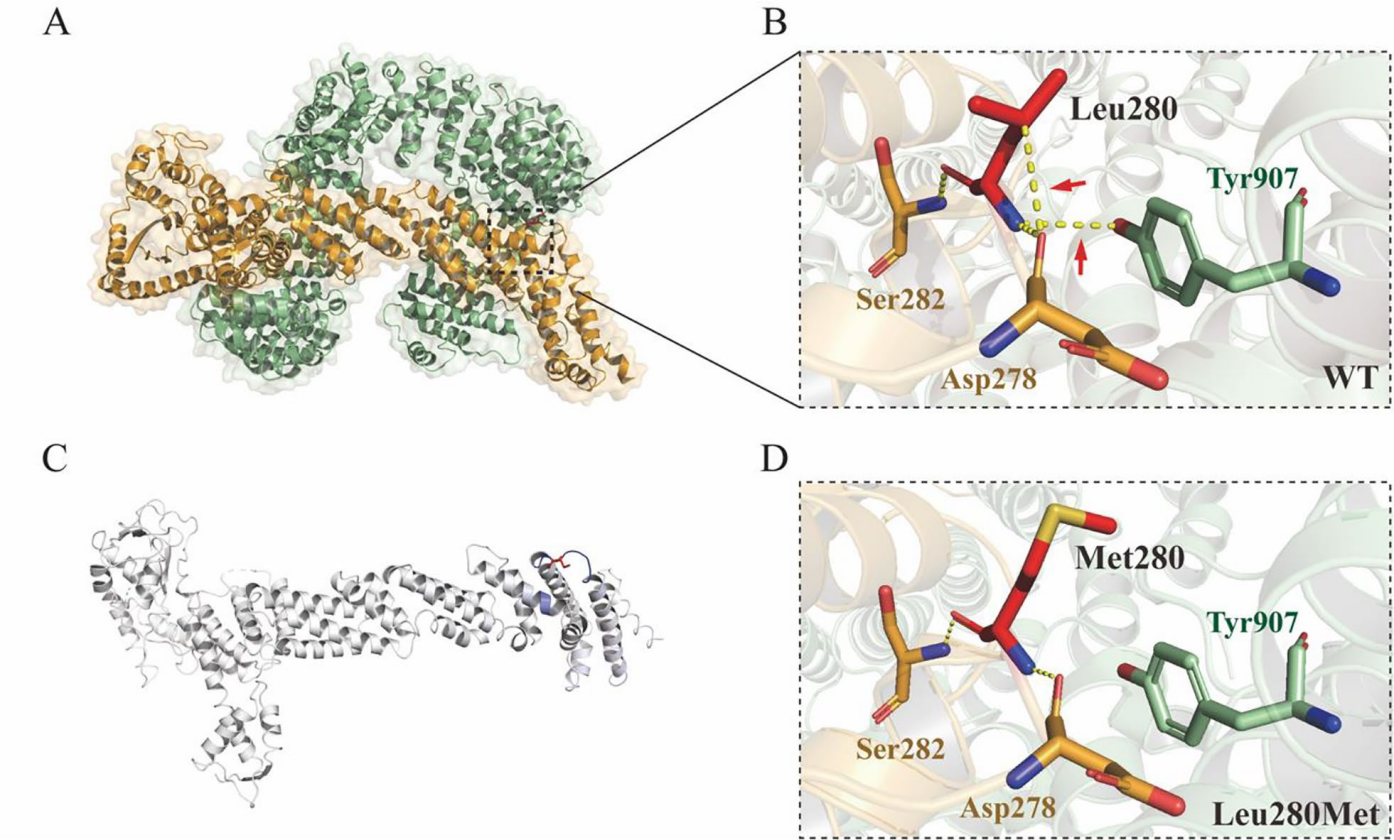
Three-dimensional structure analysis and the protein dynamics of CUL4B. **A** Three-dimensional structure of CUL4B protein and its interacting protein CAND1. The black dotted box marks the position of the Leu280Met variant. **B** Prediction of interactions between amino acid residues of wild-type. Wild-type residue is represented with red sticks. These are placed alongside surrounding residues, which are also involved in other types of interactions. Red arrows indicate interactions between altered amino acids. Hydrogen bonding is represented with a yellow dotted line. **C** Δ Vibrational Entropy Energy | Visual representation of variant: Amino acids colored according to the vibrational entropy change of the variant. Blue represents a rigidification of the structure. **D** Prediction of interactions between amino acid residues of Leu280Met variant. Leu280Met residue is represented as red sticks

The core pedigree members of 46, XY DSD, including her parents, were sequenced using trio-WES and Sanger (Fig. 1C). Within the exome region, there were 27,739 variants, with 65.15% being nonsynonymous (Fig. 1D). After filtering, the 1441 nonsynonymous variants were reduced to 11 rare variants. Finally, a variant site was identified in the *CUL4B* gene (c.838 T > A) (Fig. 2E). The Sanger sequencing indicated that the proband carried the c.838 T > A (p.L280M) hemizygous variant, and her mother had a heterozygous c.838 T > A variant of the *CUL4B* gene. Additionally, WES and Sanger sequencing confirmed a missense variant of the *CUL4B* gene, which was not present in the public population database gnomAD (https://gnomad.broadinstitute.org/), Chinese Millionome Database (https://db.cngb.org) or Human Gene Mutation Database (http://www.hgmd.org/) [18].

To further analyze the genetic testing data, we evaluated the interspecific conservation and pathogenicity of the c.838 T > A variant. We compared the CUL4B protein sequences of representative vertebrates, including *Homo sapiens*, apes, and mice (Fig. 1F). Leu280 residues in *CUL4B* exhibited a high conservation level among all vertebrates (100%) (Fig. 1F).

### Three-dimensional structure modeling

The impact of the identified mutations on the protein shape was examined using the 3D structure of human *CUL4B* (PDB code 4A0C) to determine the structural alterations in *CUL4B*. The DynaMut webserver was used to analyze and visualize protein dynamics and determine the effects of the variant. The DynaMut web server was also used to evaluate the local interactions among wild-type and variant amino acid residues. In both the PDB file and in the Protein Interfaces Surfaces and Assemblies (PISA server, https://www.ebi.ac.uk/pdbe/pisa/), Leu280 residue was found to be involved in a multimer contact. The PISA database contains protein assemblies with a high probability of being biologically relevant. This strongly suggests that the residue is in contact with other proteins (Fig. 2A, B). Structural protein analysis through the HOPE protein analysis server indicated that the Leu280Met variant introduces a bigger residue at this position, which can disturb the multimeric interactions. The p.L280M variant also decreases molecule flexibility (ΔΔSVibENCoM: –0.146 kcal.mol^–1^.K^–1^) (Fig. 2C). Furthermore, the data indicated that this variant resulted in the conversion of leucine (Leu) to methionine (Met), which not only reduces hydrogen bonding with Asp287 but also disrupts its interaction with Cullin-associated NEDD8-dissociated protein 1 (CAND1) (Fig. 2D).

### ***CUL4B***^L280M^ leads to hyperactivation of Wnt4/β-catenin signaling pathway

**Fig. 3:**
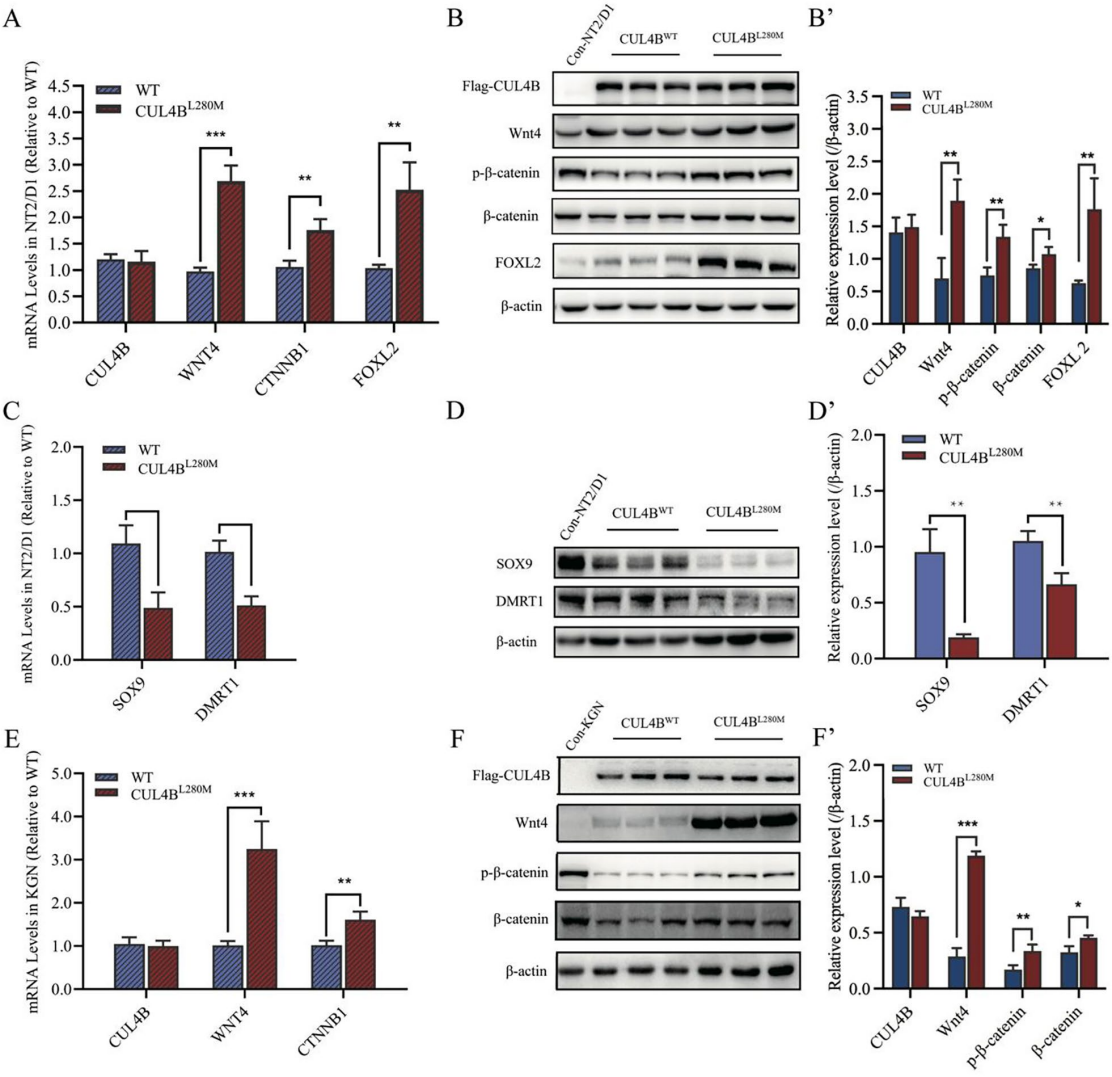
Upregulated expression of ovary pathways. **A**–**D** NT2/D1 cells transfected with empty vector plasmids expressing either wild-type (WT) or mutant (CUL4B^L280M^) *CUL4B* genes. **A** mRNA levels of *CUL4B*, *WNT4*, *CTNNB1,* and *FOXL2* measured using quantitative PCR. **B** Western blot analysis of CUL4B, Wnt4, p-β-catenin, β-catenin and FOXL2. (**B**′) Quantified analysis of western blots of CUL4B, Wnt4, β-catenin, and FOXL2. **C** mRNA levels of *SOX9* and *DMRT1* were measured through quantitative PCR. **D** Western blot analysis of SOX9 and DMRT1. (**D**′) Quantified analysis of western blots of SOX9 and DMRT1. **E** and **F** KGN cells transfected with empty vector plasmids expressing either wild-type (WT) or mutant (CUL4B^L280M^) *CUL4B* genes. **E** mRNA levels of *CUL4B*, *WNT4,* and *CTNNB1* in KGN cells were measured using quantitative PCR. **F** Western blot analysis of CUL4B, Wnt4, p-β-catenin, and β-catenin. (**F**′) Quantified analysis of western blots of CUL4B, Wnt4, p-β-catenin, and β-catenin. For western blotting, results were normalized to β-actin as a reference (n = 3; mean ± SD; *p < 0.05, **p < 0.01 and ***p < 0.001)

We examined how the *CUL4B*^L280M^ variant impacts sex-specific signaling pathways by investigating its influence on the expression of key genes associated with testicular and ovarian development. The wild-type or mutant *CUL4B* gene in the NT2/D1 testicular teratoma cells line and KGN ovarian cells was overexpressed by lentiviral infection. We observed no significant difference between the wild-type and mutant groups in *CUL4B* mRNA and protein expression. In NT2/D1 cells, the mRNA expressions of *WNT4* in the mutant group were 2.6 folds higher compared with the wild-type group (Fig. 3A). Moreover, the protein expressions had increased to 2.8 folds of those of the wild-type (Fig. 3B and B′). The mRNA expressions of *CTNNB1* in the mutant group were 1.7 folds higher than those in the wild-type group. The p-β-catenin and β-catenin protein expressions increased to 1.8 and 1.3 folds of the wild-type, respectively (Fig. 3A, B). The mRNA and protein expression of *FOXL2* in the mutant group was 2.4 and 2.7 folds higher, respectively, than in the wild-type group (Fig. 3A, B, and B′). Then, we analyzed the expression of key genes, including *SOX9* and *DMRT1,* for testis development. The mRNA expressions of *SOX9* and *DMRT1* in the mutant group were 44.7% and 50.7% of those in the wild-type group (Fig. 3C). The protein expressions were reduced to 19.9% and 63.0% of the wild-type, respectively (Fig. 3D and D′).

Additionally, the mRNA levels of *WNT4* and *CTNNB1* in KGN ovarian cells of the mutant group were 3.19 and 1.58 folds of the wild-type group, respectively (Fig. 3E). The mutant group exhibited increased Wnt4, p-β-catenin, and β-catenin protein levels of 4.0, 1.9, and 1.4 folds compared to the wild-type group (Figs. 3F and F′). These results suggested that the *CUL4B*^L280M^ variant activates the ovarian pathway (Wnt4/β-catenin) in testicular and ovarian cells.

### ***Cul4b***^L337M^ variant in mice disrupts testicular vasculature and reproductive function

**Fig. 4:**
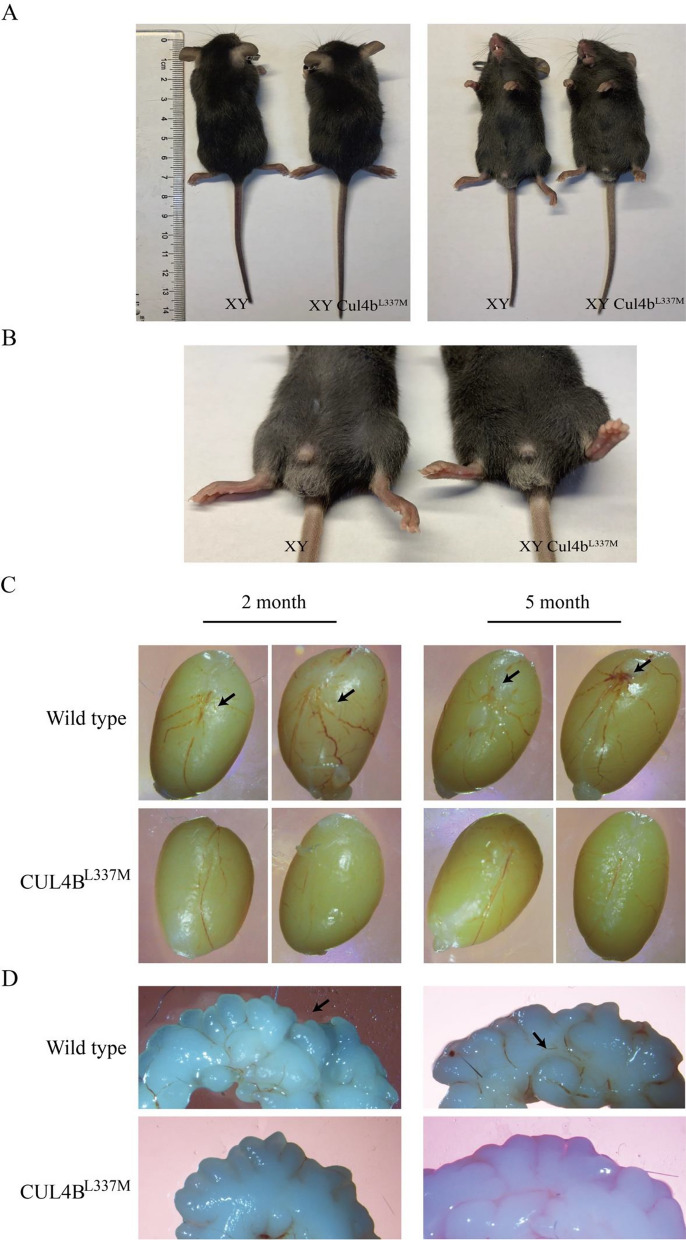
Vasculature phenotype of testis and seminal vesicles in* Cul4b*^L337M^ mice. The overall (**A**) and penises (**B**) appearance of *Cul4b*^L337M^ and wild-type mice. **C** Vasculature of testis in wild-type and *Cul4b*^L337M^ mice (2 and 5 months of age). The black arrow points to the testicular artery in wild-type mice. **D** Appearance of seminal vesicle in wild-type and *Cul4b*^L337M^ mice (2 and 5 months of age). The seminal vesicles of wild-type mice are deeply invaginated; however, mutant mice lack this characteristic

To determine the effect of the gain of the *CUL4B* gene variant on gonadal development, we generated precision-edited mice (*Cul4b*^L337M^) carrying the homologous variant of human *CUL4B*^L280M^. The overall appearance of *Cul4b*^L337M^ mice exhibited no significant difference from the control mice (Fig. 4A, B). We examined the mouse testicular vasculature (2- and 5-month-old), as this is a major feature of the testis, and found that the testicular vasculature had a disruption in male mice carrying the *Cul4b* gene variant. The testicular aortas of mutant mice were narrowed, and arterial branches were significantly reduced compared to the wild type (Fig. 4A). In *Cul4b*^*L337M*^ males, the seminal vesicles were less developed compared to the wild-type organ, as evidenced by the absence of typical deep invaginations. Additionally, the vascular distribution of seminal vesicles of mutant mice was significantly less than that of wild-type mice (Fig. 4B).

**Fig. 5:**
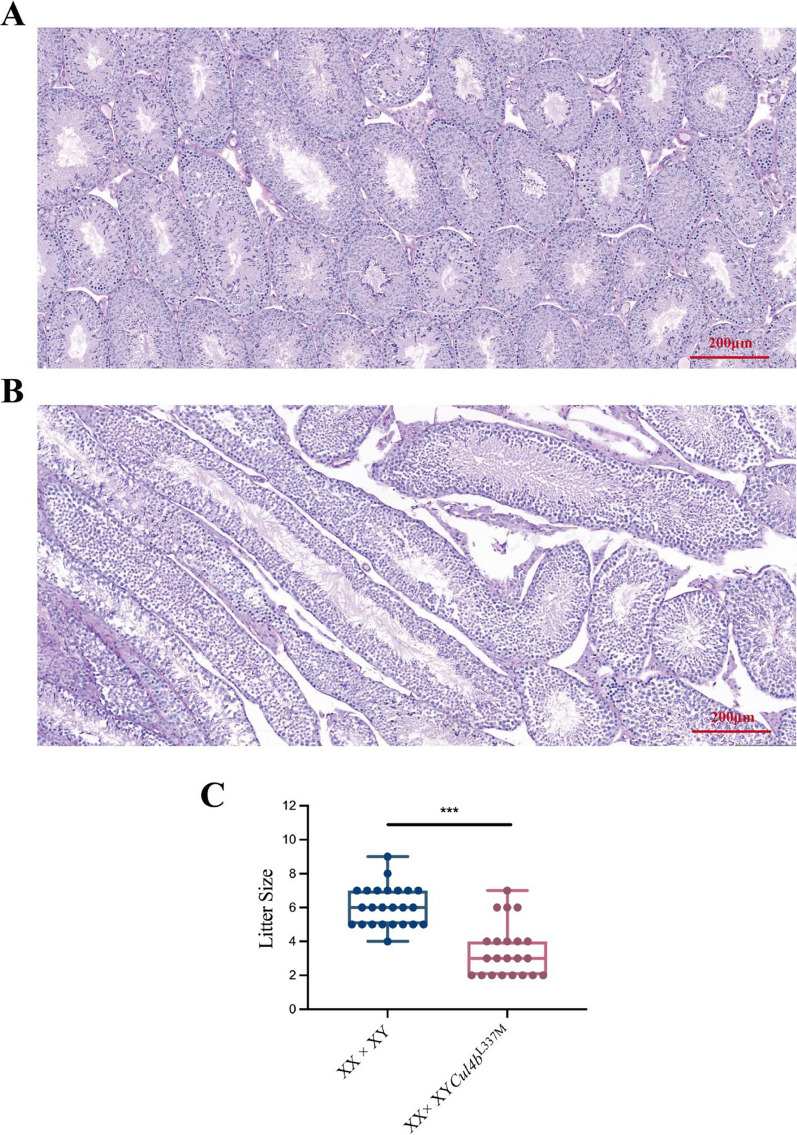
Testicular pathology and fecundity in mice. **A**, **B** Testis by hematoxylin–eosin (HE) staining. **A** HE staining of testis of wild-type mice. **B** HE staining of testis of *Cul4b*^L337M^ male mice. **C** The numbers of pups per litter of wild-type mice and *Cul4b*^L337M^ mutant male mice crossed with wild-type female mice (n > 20; mean ± SD; *p < 0.05, ** p < 0.01 and ***p < 0.001)

Furthermore, HE staining of testis tissue of mutant male mice exhibited elongation of seminiferous tubules, thinning of the epithelium, atrophy of the seminiferous tubules, and a modest reduction in round spermatids (Fig. 5A, B). These features were consistent with Wnt4 transgenic males. We determined fertility in males carrying the *Cul4b* gene variant (n > 10) by performing test crosses to wild-type female mice. At 2-month-old, the fecundity of wild-type mice (6.08 ± 1.15 neonatal mice per litter) was significantly higher than that of *Cul4b*^L337M^ mutant male mice (3.45 ± 1.53 neonatal mice per litter), *p* < 0.001 (Fig. 5C).

## Discussion

The *CUL4B* gene is an important cullin-Ring E3 ligase gene family member. The *CUL4B* gene is located in Xq23 and consists of 22 exons with a full mRNA length of 5785 bp. *CUL4B* plays an important role in the cell cycle, embryo development, and spermatogenesis through ubiquitin-dependent protein degradation and epigenetic regulation. The *CUL4B* gene has been reported to cause intellectual developmental disorder, X-linked, syndromic, Cabezas type (OMIM#300354). The main clinical manifestations of *CUL4B* gene variant patients are neurological abnormalities, short stature, obesity, and reproductive tract abnormalities [19–21]. Reproductive tract abnormalities include cryptorchidism and/or small testes, hypospadias, micropenis, and spermatogenesis abnormalities [19, 22]. Here, we describe a patient presenting with 46, XY female DSD in a Chinese family. WES and Sanger sequencing identified a hemizygous missense variant c.838 T > A (p. Leu280Met) in the *CUL4B* gene. Segregation analysis in the family revealed that the affected patient had inherited the variant from her healthy mother (heterozygous), while the father and brother did not carry the variant. The *CUL4B* c.838 T > A variant was not identified through a comparison with population data from the Chinese Millionome Database (db.cngb.org/cmdb/). Moreover, the patient exhibited no significant neurological abnormalities. For the *CUL4B* gene, the c.838 T > A variant causes an amino acid change from leucine (Leu/L) to methionine (Met/M). Evolutionary conservation analysis revealed that the L280 residue was highly conserved among species. Furthermore, the PISA database and DynaMut online server predicted that there was a significant alteration in the local structure of the p.L280M variant. The Leu280Met variant introduces a larger residue at this position, which can interfere with the multimeric interactions and reduce molecule flexibility. This evidence supports the likely pathogenic nature of the Leu280Met variant. *CUL4B* is an E3 ubiquitin ligase that interacts with diverse substrates and ubiquitinates them. In addition to *CAND1*, several proteins interact with *CUL4B*; however, the mode of protein–protein interaction remains unknown.

Multiple genes are expressed simultaneously throughout human gonadal development [23, 24]. *SRY* is active in the somatic gonads of XY individuals during sex determination, resulting in an increase of *SOX9* and the initiation of testis development [25]. Ovarian differentiation is promoted without *SRY* (XX individuals) by activating WNT/CTNNB1 through upregulating *Rspo1* and *Wnt4* [26]. Furthermore, in the human embryonic period, different sex-determining factors, including DMRT1 (OMIM*602424) and FOXL2 (OMIM*605597), are essential for regulating the formation and maintenance of either testicular or ovarian pathways [27–29]. The bipotential gonads’ differentiation is a critical phase in forming an embryo’s identity. A balance between transcriptional activation and repression determines this destiny specification. The *CUL4B*^L280M^ variant in NT2/D1 cells resulted in increased expression of genes associated with ovarian development (including *WNT4*, *CTNNB1*, and *FOXL2*) and decreased expression of testicular development-related genes (*SOX9* and *DMRT1*). Studies have reported that *CUL4B* activates Wnt/β-catenin signaling by repressing Wnt antagonists [30–32]. However, the effect of *CUL4B’s* interactions with its proteins on the Wnt/β-catenin pathway remains unclear. We hypothesized that ubiquitination could be associated with DSD.

WNT4 is a secretory protein essential for female sex development because it inhibits male sexual differentiation in humans [33, 34]. The Wnt4/β-catenin pathway is critical for the formation of ovaries during embryonic development in both mice and humans [35, 36]. Mutations in *Wnt4* or *Ctnnb1* in mouse XX gonads lead to the gradual development of ovotestes, exhibiting features of both testes and ovaries [35, 37]. Abnormal initiation of the WNT/β-catenin pathway or *FOXL2* in XY reproductive organs results in reduced *SOX9* expression, which can lead to the development of ovaries [35, 38–40]. Copy number gain or gain of function of the *WNT4* gene in humans results in varying degrees of 46, XY DSD, with clinical manifestations ranging from severe complete gonadal dysgenesis to mild phenotypes including hypospadias, cryptorchidism, and micropenis [40, 41]. In cellular experiments, we demonstrated that the *CUL4B*^L280M^ variant activates the Wnt4/β-catenin/FOXL2 signaling pathway [42]. Next, we tested our hypothesis using the precision-edited mice *CUL4B*^L337M^ model. In the present study, the male mouse model exhibited that the *CUL4B*^L337M^ variant interferes with the normal development of male gonadal vasculature and testosterone biosynthesis.

Additionally, the seminal vesicles of CUL4B males were underdeveloped and lacked the deep invagination characteristic of wild-type organs. Testicular histology exhibited spermatogenic tubule elongation, epithelial thinning, and moderate reduction of round sperm cells. These characteristics are consistent with the phenotype of *WNT4* transgenic male mice [41]. Therefore, our results suggested that *CUL4B* gene variation can be involved in the pathogenesis and development of 46, XY DSD through abnormal activation of the Wnt4/β-catenin pathway. Moreover, the *CUL4B* gene transcript is highly expressed in placental cells, embryonic stem cell lines, and testes in mice [42]. Human *CUL4B* is highly expressed in pancreatic tissue, endocrine glands, cerebellum, digestive tract, bone marrow, and testes [43, 44]. *Cul4b*-deficient mice experience growth cessation and degeneration during the initial embryonic phases and die at E9.5, while our genetically modified mice remain unaffected [45, 46]. Consistent with our patient phenotype, our mice lacked neurological manifestations, indicating that the *Cul4b*^L337M^ variant is tolerable for neurological development. The mouse model exhibited a relatively mild gonadal phenotype, with no sex reversal individuals. This could be due to differences between the murine and human systems regarding sex determination or sex differentiation. Moreover, it is necessary to collect additional gonadal phenotypes of patients with *CUL4B* gene variation to further clarify the correlation between genotype and phenotype.

In conclusion, we have outlined the clinical features of a proband exhibiting 46, XY female DSD caused by a missense mutation in the *CUL4B* gene (c.838 T > A/p.L280M). Experiments on in vitro and in vivo animal models indicated that *CUL4B* gene variation is associated with 46, XY female DSD, which can be caused by activation of the Wnt4/β-catenin pathway. Our findings provide new insights into the clinical evaluation and molecular basis of *CUL4B*-associated 46, XY female DSD.

## Supplementary Information

Supplementary material 1.

## Data Availability

The variants have been submitted to ClinVar database [Accession numbers: SCV001737544 (CUL4B c.838 T > A: p. Leu280Met].

## Abbreviations

ACMG: American College of Medical Genetics and Genomics
CUL4B: Cullin 4B
DSD: Disorders of sex development
DMRT1: Double sex and mab-3-related transcription factor 1
FOXL2: Forkhead box protein L2
HE: Hematoxylin and eosin
HGVS: Human Genome Variation Society
*trio*-*WES*: *Trio* Whole exome sequencing
WES: Whole-exome sequencing
Wnt4: WNT family member 4
