# Supplementary material for: A novel CUL4B gene variant activating Wnt4/β-catenin signal pathway to karyotype 46, XY female with disorders of sex development
Source: Biol Res. 2025 Jan 7;58:1. doi: 10.1186/s40659-024-00583-1 (PMC11705720; doi:10.1186/s40659-024-00583-1)
Supplement: Supplementary file 1 — Supplementary material 1. [file 40659_2024_583_MOESM1_ESM.docx]

**Table S1** Primer list for qPCR

| Gene | Gene ID | NCBI Reference Sequence | Forward primers  5’→3’ | Reverse primers  5’→3’ |
| --- | --- | --- | --- | --- |
| CUL4B | 8450 | NM_003588.4 | GAAGCTACAGATGAAGAACTTGAG | GCACTCTTTCCGACTAACAGGC |
| SOX9 | 6662 | NM_000346 | AGGAAGCTCGCGGACCAGTAC | GGTGGTCCTTCTTGTGCTGCAC |
| CTNNB1 | 1499 | NM_001098209 | CACAAGCAGAGTGCTGAAGGTG | GATTCCTGAGAGTCCAAAGACAG |
| WNT4 | 54361 | NM_03C0761 | GCTGGAGAAGTGCGGCTGTGA | CCACAAACGACTGTGAGAAGGC |
| FOXL2 | 668 | NM_023067 | CGGAGAAGAGGCTCACGCTGT | CTGAGGTTGTGGCGGATGCTAT |
| DMRT1 | 1761 | NM_021951 | CCTTATGTGCCTGGTCAGACAG | GCATCCTCAAAAGAGAAAAACTGG |
| ACTB | 60 | NM_001101 | CACCATTGGCAATGAGCGGTTC | AGGTCTTTGCGGATGTCCACGT |
